# Update and validation of the Canadian Behavior, Attitude and Nutrition Knowledge Survey (C-BANKS 2.0)

**DOI:** 10.3389/fpubh.2023.1261146

**Published:** 2023-10-03

**Authors:** Lynne M. Z. Lafave, Joyce Hayek, Mark R. Lafave

**Affiliations:** Department of Health and Physical Education, Mount Royal University, Calgary, AB, Canada

**Keywords:** nutrition, questionnaire, knowledge, attitude, behavior, validation, healthy environments, community

## Abstract

**Aim:**

Understanding knowledge, attitudes and behaviors related to nutrition is crucial in developing effective intervention strategies to promote healthy eating habits. This study revised and updated the Canadian Behavior, Attitude and Nutrition Knowledge Survey (C-BANKS) to align with the current Canada’s Food Guide and dietary guidance and report on its reliability and validity with a new population.

**Method:**

Data from 167 early childhood educators were recruited to test the revised C-BANKS’ reliability and validity. Reliability, as measured by internal consistency, was assessed using Cronbach’s alpha, while concurrent validity was determined by correlating the C-BANKS 2.0 scores with the CHEERS (Creating Healthy Eating and Active Environments Survey) and Mindful Eating Questionnaire (MEQ). Responsiveness was assessed by comparing a baseline C-BANKS 2.0 score to a post-test score after completing an online healthy eating and physical activity educational intervention.

**Results:**

The adapted C-BANKS 2.0 questionnaire demonstrated good internal consistency (Cronbach’s alpha >0.70) and construct validity with related measures CHEERS and MEQ (*p* < 0.05). The C-BANKS 2.0 demonstrated strong responsiveness. Specifically, C-BANKS 2.0 scores increased after the nutrition education intervention (*p* < 0.001). Additionally, there were no signs of floor or ceiling effects.

**Conclusion:**

The adapted C-BANKS 2.0 demonstrated satisfactory internal consistency, construct validity, and responsiveness to measure of nutrition knowledge, attitudes, and behaviors in an early childhood educator population. The revised C-BANKS 2.0 provides insight into the key factors that influence dietary habits thereby informing the design and evaluation of effective nutrition community intervention programs.

## 1. Introduction

Research consistently demonstrates the link between good nutrition, improved overall health, and mental well-being (1–7). Assessing knowledge, attitudes and nutrition-related behaviors is critical in order to plan effective intervention strategies to promote healthful eating behaviors and consequently to contribute to improved health and quality of life.

Nutrition knowledge is defined as the understanding of concepts and processes related to nutrition and health, including nutrient sources, the diet and disease relationship, as well as dietary guidelines (8). Greater nutrition knowledge has been linked to increased fruits and vegetables intake (9), lower intakes of fat (10) and healthier food choices supported by food label use (8, 11). The suggested working mechanism through which nutrition knowledge influences nutrition behavior includes its effects on cognition (12). Health behavior theories assume that knowledge is an important distal factor that can support behavioral change by influencing attitudes and motivation to perform a certain health behavior (12). Sufficient levels of nutrition knowledge can influence individuals’ motivation to adopt a healthy diet and foster more positive attitudes towards healthy eating (13, 14). Nutrition knowledge is recognized as an important predictor of food choice behavior (15). Additionally, measurement of nutrition knowledge is also an important piece in understanding the complex relationship with nutrition attitudes.

According to behavior change theories, attitude is the favorable or unfavorable feeling towards a particular behavior and is considered a predictor of people’s intentions and behaviors (12, 16–18). Positive or negative attitudes towards nutrition and health have been found to influence food selection decisions and nutrition behaviors (19). Again, measuring the interplay between people’s knowledge, attitudes, and behaviors can be helpful in planning interventions that are targeted to improve health.

The Knowledge, Attitude, and Practice (KAP) model provides a framework for facilitating meaningful change in nutrition practices (20). Assessing knowledge, attitudes, and practices is the first step in the behavior change process as it will inform the development of health promotion initiatives to change those behavioral determinants and ultimately promote behavior change (21, 22). Many KAP tools exist to measure what people know, believe, and do with regards to health and nutrition. The Diet and Health Knowledge Survey (23) is one such example that has been developed for the U.S population. Validity cannot be assumed outside the population or culture for which it has been developed. Regardless of whether (24) the DHKS has demonstrated validity and is considered a comprehensive measure of nutrition knowledge and attitudes in the U.S population (25).

Little information on the knowledge, attitudes and nutrition-related behaviors of Canadians is known. Lafave et al. (26) developed the Canadian Behavior, Attitude and Nutrition Knowledge Survey (C-BANKS) to address the need for a comprehensive survey assessing dietary knowledge, attitudes, and behaviors among the Canadian adult population. Subsequently, other tools were created for the Canadian context, but these tools predominantly measure nutrition knowledge alone (27, 28) or knowledge and attitudes (29). To date, the C-BANKS is the only Canadian specific survey that measures nutrition-related knowledge, attitudes, and practices in a single questionnaire. This survey can be used to inform program and policy development, as well as evaluate the effectiveness of health behavior interventions for the Canadian adult population.

Theories of behavior change were used to develop the original questionnaire (16, 18, 30). The original C-BANKS was comprised of 132 items assessing dietary knowledge (knowledge of food guide, important nutrients, as well as nutrition and health interrelationships), attitude (perception of healthy eating) and practices (food choice behavior, use of nutrition labels). The content of this questionnaire was based on Canada’s Food Guide (CFG) at the time (i.e., 2007). Canadian experts were consulted at the development and validation stage followed by feedback on the content, sensitivity, and accuracy of the items in measuring the different constructs. A more detailed description of the original instrument design and validation is described elsewhere (26).

Canada’s dietary guidelines were revised in the updated January 2019 Canada’s Food Guide (31). The changes to the 2019 version of the CFG were significant, particularly when it is compared with the 2007 CFG version (32) on which the original C-BANKS was designed. Changes included removal of serving specific daily targets, a refocus on plate guidelines, recommendations to increase plant-based foods, guidance to limit highly processed food, and sugar-sweetened beverage intakes. The C-BANKS was originally tested on a young adult population. In the current study, the target population was early childhood educators (educator). Understanding educators’ personal nutrition knowledge, attitude and behaviors plays a crucial role in shaping children’s eating behaviors (19, 33). The revised C-BANKS (C-BANKS 2.0 herein) was administered to the educator population as part of larger research project evaluating the effect of an intervention within early learning settings. This paper will only focus on the C-BANKS revision. It is important to assess a tool in multiple populations to build validity context over time and across populations (24). Educators are part of the adult Canadian population and employing the C-BANKS within this population will test a subset of the Canadian population and add to a growing body of evidence to speak to the tool’s overall validity. Therefore, the purpose of this study is to 1) revise and update the C-BANKS to reflect the most current dietary guidelines and 2) evaluate its reliability and validity.

## 2. Methods

### 2.1. Participants

The data was sourced from a larger research project *“CHEERS HEAPful of FUN: raising healthy Albertans”* conducted between 2019 and 2021. This project evaluates the impact of a healthy eating and physical activity intervention in changing professional practices of early childhood educators. Educators complete twelve weekly online education modules and attend communities of practice sessions as part of the intervention program. The learning module intervention addresses nutrition and physical activity in the child care setting, as well as aspects of sleep, relationship with parents, and self-care of educators. Learning modules were supplemented with online community of practice sessions so as to provide the educators with an opportunity to share their personal experiences and deepen their understanding within a learning community.

Educators were recruited from two metropolitan and two mid-sized cities across Alberta, Canada. Educators were eligible to participate if they were part of an ECEC center that was a licensed facility-based center that served children between 2 and 5 years, had internet connection, and were not currently enrolled in any other intervention to improve nutrition and activity practices. Center directors were contacted by phone and provided with information on the study. If center directors agreed, a project coordinator recruited educators from a center. Those who agreed to take part in the study received an email package with instructions, a consent form, survey links and contact information of a trained research associate to answer questions that arose. This study followed the ethical guidelines laid down in the Declaration of Helsinki (34) and the Mount Royal University Human Research Ethics Board approved the study protocol (no. 101768).

Data was collected from 167 educators at two time points, pre- and post-intervention after 10 months. Educators completed a baseline (pre-test) evaluation of their nutrition knowledge, attitudes, and behaviors with the C-BANKS 2.0, followed by online educational modules, and finally, followed by a post-test C-BANKS 2.0 test. The learning module intervention addressed nutrition and physical activity in the childcare setting, sleep, relationship with parents and self-care of educators. Educators attended weekly community sessions facilitated by early childhood education experts to reflect on the practical applications of the module topics within their childcare programs.

Educators also completed demographic questions, the CHEERS (Creating Healthy Eating and active Environments Survey), and the Mindful Eating Questionnaire (MEQ). All surveys were completed online using the Qualtrics^©^ survey platform.

### 2.2. Instruments

The primary instrument of interest in this study was the C-BANKS 2.0. However, other instruments were also employed to contribute to the validation of the C-BANKS 2.0. A short description of the revised version of the C-BANKS 2.0 is outlined followed by a description of the other instruments that were employed to measure longitudinal validity.

#### 2.2.1. C-BANKS

The original C-BANKS was developed as a Canadian context specific tool and consistent with Canada’s Dietary Guidelines. The survey was based on the 2007 Canada Food Guide, literature review, and nutrition expert consultations. In the current study, the C-BANKS was revised to ensure alignment with the most current evidence and reflect compliance with the updated Canadian Guide released in 2019. The revisions included changes relating to wording of some items, deletion of outdated questions and addition of new questions. An example where a large number of items requiring revision included the knowledge of serving sizes (e.g., ½ cup vegetables) and number of servings per person based on age and sex. In the 2019 CFG the knowledge item became half a plate of vegetables at each meal. Details of revised items are provided in the Supplementary material S1. The C-BANKS 2.0 consists of a total of 60 items, 20 items assessing knowledge, 5 assessing attitude and 35 behavioral questions.

##### 2.2.1.1. Knowledge construct

Nutritional knowledge questions focus on measuring factual information and participant’s awareness of specific nutritional concepts such as the link between nutrition and health (e.g., *“Which of the following is a health problem related to eating added sugars?”*). The knowledge subscale which originally included 40 questions was shortened to 20 questions to align with 2019 CFG update. The scoring remained the same with a correct response given a score of +1, whereas incorrect or unknown answers scored 0. The sum of all items provided the total score and a higher scoring indicated better knowledge.

The original C-BANKS assessed knowledge about food group intake on the basis of the number of servings. The new Food Guide does not include specific recommendations regarding serving sizes but rather relies on relative proportions instead of weight and volume. In addition, the new guide promotes shifting intakes towards more plant-based foods and shows half of the food coming from fruits and vegetables, a quarter from whole grain products and the remaining quarter from protein foods (31). Therefore, in the C-BANKS 2.0, questions asking about specific serving sizes for food groups were updated to the current recommendations. For example, the question “*How many servings from the following Canada’s Food Guide groups should a person of your age and sex eat each day for good health?”* was replaced with *“Which is the best option for serving a healthy plant-based meal?*”

A new question “*Which of the following foods would be considered a highly processed food?*” was included to reflect the new guidelines of limiting intakes of processed meats and foods high in saturated fats.

The new Food Guide highlights the importance of using food labels to make informed food choices and provides guidance for meeting requirements of essential nutrients. The questions on nutrients and food labels were thus modified in the C-BANKS 2.0 to comply with the new recommendations (e.g., *“Which food is a good source of calcium?,” “Compared to a regular product, a product with the word “light” on the label may be ___?”*).

Another change made to the knowledge subscale pertains to the phrasing of some questions. Open-ended questions were replaced with multiple choice questions since the latter have been proven to be more objective, reliable, and time-efficient and facilitate analysis and information processing (35). Details of all revised knowledge items are listed in the Supplementary material S1.

##### 2.2.1.2. Attitude construct

Attitude questions were designed to gauge respondents’ beliefs and perceptions related to healthy dietary practices and their attitude towards food choices (e.g., “*What you eat can make a big difference in your chances of developing a chronic disease such as heart disease*”). The attitude subscale was shortened from 26 to 5 questions. All attitude items were modified to have a similar Likert-response format with answering options ranging from 1 = Strongly disagree to 7 = Strongly agree. Some of the changes to the attitude construct include:

The question “*A healthy diet means choosing empty calorie foods less often*” was replaced with “*A healthy diet means choosing highly processed foods less often*” to be consistent with the updated Canadian dietary guidelines that recommends limiting highly processed food and eating them less often and in smaller amounts (31).

Some questions were removed as they were deemed opinion questions or addressing misinformation, rather than measuring attitude (e.g., “*Recommendations on healthy ways to eat change so often, it’s hard to know what to believe*”).

The total attitude score was calculated as the mean of items, a higher score reflected more positive attitudes. The revised attitude items can be seen in the Supplementary material S1.

##### 2.2.1.3. Behavior construct

The behavior subscale was reduced from 55 to 35 items, with the majority of questions scored on a 7-point Likert scale “Never-Always.”

Questions that are not aligned with the updated Food Guide recommendations (e.g., “*When making food choices how often do you: Eat a variety of foods from each of the food groups daily/ Eat fish two times per week*”) were removed.

Questions about food containing trans-fat were removed, since Health Canada has banned artificial trans-fat in 2018 and manufacturers were given until 2020 to ensure it was eliminated from the food supply (e.g., “*When making food choices how often do you choose foods low in trans-fat*”).

Questions about looking at Vitamin C and A on food labels were removed since those nutrients are no longer required to be listed on the nutrition facts table because most Canadians get enough of these nutrients in their diets (36). Food label questions focused on core nutrients such as iron, calcium, sodium, fiber, saturated fat and sugar.

Based on the Canadian movement and activity guidelines adults need to be active at least 150 min per week, hence the open-ended question *“According to Canada’s Food Guide recommendations, how many moderate physical activity minutes should be accumulated per day to maintain good health for a person of your age?”* was rephrased and converted into a multiple choice question *“How many ‘sweat a little’ to ‘sweat a lot’ physical activity minutes do you accumulate in a week?”* to make it easier to measure participant’s self-reported engagement in MVPA.

The total behavior score is calculated using the average of the items. Components of the behavior subscale are presented in the Supplementary material S1.

Other questions relating to personal practice (e.g., *“Which, if any, of the following supplements do you take on a weekly basis?*) opinion questions (e.g., *“In order to create a healthy diet, indicate the importance of eating the following meals”*) and validation questions (e.g., *“How many servings would you estimate that you typically consume from each of the following food groups?”*) were not included as they did not specifically relate to nutrition-related behaviors. All revised items are presented in the Supplementary material S1.

#### 2.2.2. CHEERS

The CHEERS survey is a community-based audit tool that measures the nutrition and physical activity environment in ECEC centers. This tool has been assessed for reliability and validity with early childhood experts and educators (37–39). The CHEERS tool includes 59 items that measure four constructs: food served (23 items), healthy eating environment (18 items), healthy eating program planning (6 items), and physical activity environment (12 items). Items are measured using a 7-point scale with responses ranging from 1 = “always” to 7 = “never.” The four subscale scores are calculated using an average of the items in the grouping. The CHEERS score is calculated by a cumulative average of the four subscales (range 4–28). In the present study, only the nutrition subdomains “food served” and “healthy eating environment” of the CHEERS survey were used in the analysis.

#### 2.2.3. MEQ

The Mindful Eating Questionnaire (MEQ) has been assessed for reliability and validity to assess the mindful eating in healthy adults (40). The MEQ is a 28-item self-reported questionnaire that measures five domains of mindful eating: (1) Disinhibition (8 items), (2) Awareness (7 items), (3) External cues (6 items), (4) Emotional response (4 items) and (5) Distraction (3 items). Items are scored on a 4-point Likert scale from 1 = “never/rarely” to 4 = “always/usually,” where higher scores reflect more mindful eating. Each subscale score was calculated as the means of items, excluding the “not-applicable” responses. The total score was calculated as the mean of the five subscales.

### 2.3. Instrument property assessment

#### 2.3.1. Reliability measures

Cronbach alpha, as a measure of internal consistency, was calculated for the overall C-BANKS 2.0 score and each subscale using data from the survey submissions. Mokkink and colleagues cluster internal consistency within the domain of reliability such that it provides an understanding of the interrelatedness of a tool’s assessment items (41).

#### 2.3.2. Validity measures

The knowledge, attitude, and behavior subscales of C-BANKS 2.0 were examined for floor or ceiling effects. Terwee et al. (42) describe that floor and/or ceiling effects within an assessment tool signify missing ranges in the lower or upper end of the measurement scale, which suggests limited content validity.

C-BANKS 2.0 scores were correlated to scores on nutrition related behavior and attitude questionnaires within the Creating Healthy Eating and Active Environments Survey (CHEERS) as well as the Mindful Eating Questionnaire (MEQ). Construct validity refers to the degree to which the scores of an instrument are correlated with scores of other instruments that align with the hypothesized outcome of the construct to be measured (41, 42).

#### 2.3.3. Responsiveness

Responsiveness was assessed by determining the change in C-BANKS 2.0 scores pre- and post-intervention. Responsiveness refers to the ability of an instrument to detect changes over time in the construct measured (41, 42). Terwee et al. (42) describe this as a measure of longitudinal validity that speaks to the validity of a change score.

### 2.4. Data analysis

Data were analyzed using IBM SPSS Statistics for Windows, version 28 (Armonk, NY, United States: IBM Corp.) and *p* value <0.05 was considered to be significant. Mean and standard deviations (SD) were used to present numerical variables, whereas numbers and percentages were used for categorical variables. Cronbach’s alpha was used to measure the internal consistency reliability of the C-BANKS subscales and global questionnaire. The knowledge, attitude and behavior subscales were examined for floor and ceiling effects. The percentage of participants reporting the lowest score (0 for knowledge and 1 for attitude and behavior) and the highest scores (20 for knowledge and 7 for attitude and behavior) was calculated. Floor and ceiling effects were considered present if >15% of participants had scored the highest and lowest scores, respectively (42). Construct validity was assessed by investigating correlation between C-BANKS 2.0 scores to scores of other related instruments (41). Data were tested for normality using the Shapiro–Wilk test. Since the data were not normally distributed, a Spearman’s correlation was run to assess the relationship between the C-BANKS 2.0, MEQ and CHEERS. Responsiveness was assessed with paired-samples t-test to determine whether there was a statistically significant mean difference in the C-BANKS 2.0 between pre- and post-intervention. *p*-values were adjusted for multiple testing using the Benjamini-Hochberg method (43).

## 3. Results

### 3.1. Characteristics of study population

Of the 177 centers contacted, 83 center directors (47%) agreed to allow recruitment of educators in their center. Eight centers withdrew from the study once the intervention began for three primary reasons, change in center director, center closed, and center staff left the profession resulting in 75 centers total. Fifty of these centers (66.7%) were privately operated and 25 (33.3%) were not-for-profit centers. Of the 225 potential educator participants being recruited from the 75 centers, 167 agreed to participate (74%). A subset of 50 participants completed the C-BANKS survey at pre- and post-intervention. Demographic characteristics of the educators are presented in Table 1. The majority of participants were females (94.6%) with a mean age of 40.5 years. The educational level achieved was predominantly the two-year diploma CDS (46.9%) followed by a university degree (26.3%) and the remaining achieving the orientation course (15%) or one-year certificate (11.9%).

**Table 1:** Characteristics of ECEC educators.

Characteristics	Frequency (%)
ECEC educator	
Highest education achieved	
CDA – Orientation Course	24 (15)
CDW – 1-year certificate – ECEC	19 (11.9)
CDS – 2-year diploma – ECEC	75 (46.9)
University degree	42 (26.3)
Age (yrs)	40.5 ± 11.98
Gender	
Male	7 (4.2)
Female	157 (94.6)
Position	
Director	57 (35.6)
Educator	90 (56.3)
Cook/Chef	2 (1.3)
Owner/Operator	11 (6.9)

### 3.2. C-BANKS 2.0 internal consistency

The C-BANKS 2.0 demonstrated good psychometric properties in the Canadian educator population. The C-BANKS total score reported a good Cronbach’s alpha (0.93) for reliability. Each subscale demonstrated satisfactory internal consistency reliability: Knowledge (0.62), Attitude (0.72), Behavior (0.94).

### 3.3. C-BANKS 2.0 floor and ceiling effects

For all the subscales, the proportion of responses at the floor and ceiling was below 15%. No ceiling and floor effects were detected (Table 2).

**Table 2:** Floor and ceiling effects.

	Floor effect % (*N*)	Ceiling effect % (*N*)
Knowledge	0% (0)	0% (0)
Attitude	0.6% (1)	4.8% (8)
Behavior	0% (0)	0% (0)

A threshold of >15% is defined as floor or ceiling effect.

### 3.4. C-BANKS 2.0 construct validity

As shown in Table 3, there was a statistically significant, small positive correlation between the C-BANKS 2.0 and MEQ, r_s_ = 0.269, *p* = 0.002. The C-BANKS 2.0 was also significantly correlated to the nutrition subdomain of the CHEERS, r_s_ = 0.156, *p* = 0.049. Additionally, ECEC staff with higher knowledge and a more positive nutrition-related attitude had significantly better dietary behaviors (*p* < 0.05).

**Table 3:** Correlations between C-BANKS 2.0 constructs with CHEERS and MEQ.

	C-BANKS	Knowledge	Attitude	Behavior	CHEERS	MEQ
C-BANKS	–					
Knowledge	0.707*	–				
Attitude	0.691*	0.263*	–			
Behavior	0.687*	0.258*	0.290*	–		
CHEERS	0.156*	0.031	0.007	0.428*	–	
MEQ	0.269*	0.122	0.251*	0.268*	0.100	–

*Correlation is significant at the 0.05 level (2-tailed).

### 3.5. C-BANKS 2.0 responsiveness

Table 4 presents the results of the paired-samples t-test conducted to compare C-BANKS scores at pre- and post-intervention. A comparison of the mean for C-BANKS before and after the intervention shows that there is a statistically significant difference. Participants in the intervention group had a statistically significant increase of 4.93, *t* (49) =4.373, *p* < 0.001 in the total C-BANKS score at 10-month follow up. Knowledge, attitude, and dietary behavior subscale scores also improved significantly (*p* < 0.05).

**Table 4:** Change in C-BANKS 2.0 scores pre- and post-intervention.

Paired sample		*N*	Mean ± SD	Mean difference	*p*-value^†^	Effect size (Cohen’s d)
C-BANKS total (%)	Pre	50	69.09 ± 10.42	4.93 ± 7.98	<0.001	0.618
	Post	50	74.02 ± 11.13			
Knowledge	Pre	50	11.02 ± 3.38	1.18 ± 3.39	0.023	0.348
	Post	50	12.20 ± 3.76			
Attitude	Pre	50	5.68 ± 1.22	0.34 ± 1.06	0.028	0.319
	Post	50	6.02 ± 0.98			
Behavior	Pre	50	4.97 ± 1.05	0.28 ± 0.52	<0.001	0.539
	Post	50	5.25 ± 1.12			

SD, Standard Deviation.

^†^Adjusted *p*-value using the Benjamini-Hochberg method.

## 4. Discussion

The original C-BANKS development and its initial validity and reliability have been previously established and described elsewhere (44). In the content validation process of the original C-BANKS, a modified Ebel procedure was employed. Ten experts from across Canada reviewed the survey assessing the importance of each item. Items achieving an 80% consensus were included, and items not achieving 80% were brought to face-to-face discussions. During these discussions, new nutrition-related concepts were identified as important, leading to the incorporation of additional items. At the end of the process, final expert consensus was reached on all scale items providing a measure of content accuracy for the C-BANKS tool. The final questionnaire demonstrated face and content validity and a satisfactory Cronbach’s α value of 0.75.

Evidence-based dietary guidance changes over time, and therefore it is important to revise nutrition surveys based on those latest guidelines and research. The current study updated the C-BANKS with the current Canadian’s dietary recommendations and further validate the resulting C-BANKS 2.0. It demonstrated a suite of validity and reliability measures on the educator population adding to the C-BANKS overall validation. The C-BANKS 2.0 showed good internal consistency, which is an indication of a unifying construct related to nutrition knowledge, attitude, and behaviors. In addition to the C-BANKS 2.0 demonstrating strong reliability as a whole, it also demonstrated good reliability indices for the knowledge, attitude, and dietary behavior subscales. There was no evidence of floor or ceiling effects, suggesting that the C-BANKS 2.0 is suited to detect improvement and worsening in nutrition knowledge, attitude, and behaviors over time.

The paired t-test demonstrated the longitudinal validity of the C-BANKS 2.0. Responsiveness is a tool’s sensitivity to detect change over time in the constructs to be measured (41). Each of the three subscales and the total C-BANKS score were sensitive to change after an educational intervention. The significant improvement in the C-BANKS scores provides further evidence of overall validity and reliability of the revised C-BANKS. In summary, the C-BANKS 2.0 demonstrated sensitivity to changes in knowledge, attitude, and dietary-related behaviors after nutrition interventions with an early childhood educator population.

The CHEERS and MEQ instruments have demonstrated validity and reliability previously (37–40). Both questionnaires measure similar constructs to the C-BANKS. The nutrition subdomain of the CHEERS assesses healthy eating environment including feeding practices, while the MEQ measures eating behaviors focusing on the emotional and bodily experiences related to eating. The positive significant correlation of the C-BANKS 2.0 with the MEQ and CHEERS provides evidence of concurrent validity for the revised C-BANKS and indicate that they measure similar constructs. The lack of perfect correlation demonstrates both tools measure similar and unique properties.

The positive correlation between knowledge-attitude, attitude-behavior and knowledge–behavior reaffirms the interconnection of the three constructs as assumed by the KAP model (44). Our results are in line with previous research showing that greater nutrition knowledge is positively associated with favorable attitudes towards healthy eating (14, 45). Furthermore, our results confirm existing evidence of the influence of knowledge and attitude on dietary behavior. In the current study, participants with higher knowledge and favorable attitudes had higher nutrition behavior scores. This is in line with previous research showing that better dietary knowledge and more positive attitudes towards healthy eating were associated with healthier eating habits (46–49). Consequently, the C-BANKS 2.0 can be used with other populations as a baseline measure to understand determinants of dietary behaviors, guide the design of public health nutrition interventions, and evaluate the effectiveness of those interventions to further the instrument development.

### 4.1. Strengths and limitations

Some limitations should be considered when interpreting the findings of this study. Firstly, the study sample population was limited to the province of Alberta, Canada which limits the generalizability of the findings. In addition, the majority of participants (94.6%) were women. Women represent 96% of the ECEC workforce so this sampling is reflective of the participant sample (50). Moreover, educators are merely a subset of the Canadian population and future research will need to test the C-BANKS 2.0 on wider sample population to continue to test its psychometric soundness. In addition, respondent bias is possible given that data were self-reported. Lastly, the effect sizes may have been inflated in response to small standard errors possibly connected to the sampling of one to three participants in each center. Future research looking at the effects of the intervention on the changes in C-BANKS 2.0 should consider multivariate analysis to assess for covariates.

Despite these shortcomings, several strengths exist in this study. The longitudinal design of the study allows an understanding of the degree and direction of change (51). The C-BANKS 2.0 fills a gap in tools that assess nutrition knowledge, attitude, and behaviors collectively among Canadian adults (27–29). The revised C-BANKS 2.0 remains a comprehensive tool that has been adapted to reflect the most updated scientific evidence available from Health Canada’s Food Guide 2019 (31).

Finally, knowing that good scale construction is an iterative process, factor analysis is recommended in future research to create a more condensed and efficient version of the C-BANKS 2.0. The overall Cronbach’s alpha value (0.93) supports this notion of potential redundancy and the ability to reduce the total number of items in the future.

## 5. Conclusion

The main goal of the current study was to update the C-BANKS to ensure coherence with the new Canadian Food Guide. The C-BANKS 2.0 demonstrated good reliability and validity for the overall measure and acceptable reliability for the three subscales on an early childhood educator population. The survey can be used as an overall scale or administered for a particular subscale separately. The revised version may be a useful tool to gain insight into important determinants of dietary habits and thus inform the design and evaluation of nutrition intervention programs.

## Data availability statement

The datasets presented in this article are not readily available because participants’ privacy under REB approval. Requests to access the datasets should be directed to llafave@mtroyal.ca.

## Ethics statement

The Human Research Ethics Board at Mount Royal University reviewed and approved all aspects of this study protocol (no. 101768). Written informed consent was obtained from all participants involved in the study.

## Author contributions

LL: Conceptualization, Data curation, Formal analysis, Funding acquisition, Investigation, Methodology, Project administration, Supervision, Validation, Writing – original draft, Writing – review & editing. JH: Data curation, Formal analysis, Methodology, Validation, Writing – original draft, Writing – review & editing. ML: Conceptualization, Data curation, Formal analysis, Methodology, Validation, Writing – original draft, Writing – review & editing.

## Funding

The authors declare financial support was received for the research, authorship, and/or publication of this article. This research was funded by Government of Alberta, Children Services (#ACS565189). The funder had no role in the study design, data collection and analysis, decision to publish, or preparation of the manuscript.

## Conflict of interest

The authors declare that the research was conducted in the absence of any commercial or financial relationships that could be construed as a potential conflict of interest.

## Publisher’s note

All claims expressed in this article are solely those of the authors and do not necessarily represent those of their affiliated organizations, or those of the publisher, the editors and the reviewers. Any product that may be evaluated in this article, or claim that may be made by its manufacturer, is not guaranteed or endorsed by the publisher.
