# Supplementary material for: Update and validation of the Canadian Behavior, Attitude and Nutrition Knowledge Survey (C-BANKS 2.0)
Source: Front Public Health. 2023 Oct 3;11:1261146. doi: 10.3389/fpubh.2023.1261146 (PMC10584322; doi:10.3389/fpubh.2023.1261146)
Supplement: Supplementary file 1 [file Table_1.DOCX]

Supplementary Material

# Supplementary Tables

**Table 1: Knowledge items**

| Knowledge questions Original C-BANKS | Action taken | Knowledge questions  C-BANKS short form | Notes |
| --- | --- | --- | --- |
| How many servings from the following Canada's Food Guide groups should a person of your age and sex eat each day for good health?   - Vegetables and Fruit - Grain products - Milk and alternatives - Meat and alternatives   How many servings would you estimate that you typically consume from each of the following food groups?   - Vegetables and Fruit - Grain products - Milk and alternatives - Meat and alternatives | *Revised*  *Removed* | Which is the best option for serving a healthy plant-based meal? | *No more specific recommendations regarding serving sizes for food groups.*  *The new CFG promotes consumption of plant-based foods.*  *Validation question* |
| One cup (250 mL) of juice provides how many servings from the Vegetable and Fruit group? | *Revised* | According to the Canadian Food Guide, vegetables & fruit should make up approximately ___ of your plate. | *Based on the new Canada's food guide recommendations to: Make half your plate vegetables and fruits.* |
| One cup (250 mL) of cooked pasta provides how many servings from the Grain Products group? | *Removed* | The % Daily Value (DV) on the Nutrition Facts Table can be used as a guide to how much of the nutrient is in a food product. When the label reads 5% DV for a nutrient, this lets the consumer know it contains: ____ | *No more specific recommendations regarding serving sizes for food groups*  *A new question about DV was added. The new CFG highlights the importance of understanding %DV and how it can help consumers make informed food choices.* |
| A cooked skinless boneless chicken breast weighing 170 g provides approximately how many servings from the Meat and Alternatives group? | *Removed* | Which of the following foods would be considered a highly processed food? | *No longer focusing on food groups and servings*  *The new FG encourages lower intakes of processed meats and foods high in saturated fats.*  *A new question on processed food was added accordingly.* |
| One cup (250 mL) of fortified soy beverage provides how many servings from the Milk and Alternatives group? | *Removed* | The ingredient list on a food label list ingredient in order from highest to lowest by ____. | *No longer focusing on food groups and exact portion sizes*  *A new question on food label was added. The new FG encourages reading the ingredient list to make informed food choices.* |
| Identify foods in this list which provide a good source of the following vitamins. Select all that apply. [folate/b12/A/D]   - Baked beans - Spinach - Fish - Broccoli - Oranges - Beef - Milk - Nuts | *Revised* | Which food is a good source of folate?  Which food is a good source of vitamin B12?  Which food is a good source of vitamin A? |  |
| Identify foods in this list which provide a good source of the following minerals. Select all that apply. [calcium/iron/zinc]   - Baked beans - Spinach - Fish - Broccoli - Oranges - Beef - Milk - Nuts | *Revised* | Which food is a good source of calcium?  Which food is a good source of iron? |  |
| Have you heard of any health problems related to eating too much fat?  If yes, What problems are associated with eating too much fat? | *Converted to multiple choice question* | Which (if any) of the following is a health problem related to too much saturated fat? |  |
| Have you heard of any health problems related to eating too little fibre?  If yes, What problems are associated with eating too little fibre? | *Converted to multiple choice question* | Which (if any) of the following is a health problem related to eating too little fibre? |  |
| Have you heard of any health problems related to eating too much salt (sodium)?  If yes, What problems are associated with eating too much salt? | *Converted to multiple choice question* | Have you heard of any health problems related to eating too much salt (sodium)? |  |
| Have you heard of any health problems related to consuming too little calcium?  If yes, What problems are associated with consuming too little calcium? | *Converted to multiple choice question* | Which (if any) of the following is a health problem related to eating too little calcium? |  |
| Have you heard of any health problems related to consuming too little vitamin D?  If yes, What problems are associated with consuming too little vitamin D? | *Converted to multiple choice question* | Which (if any) of the following is a health problem related to consuming too little vitamin D? |  |
| Have you heard of any health problems related to consuming too much Trans-fat? | *Removed* |  | *As of 2020 trans-fat is removed form food supply* |
| Have you heard of any health problems related to eating too much sugar?  If yes, What problems are associated with too much sugar? | *Converted to multiple choice question* | Which (if any) of the following is a health problem related to eating added sugars? |  |
| Which of the following contains more saturated fat based on equal portion sizes?   - Butter - Soft margarine - Canola oil - Do not know | *Retained* | Which of the following contains more saturated fat based on equal portion sizes?   - 15ml (1tbsp) butter - 15 ml soft margarine - 15 ml canola oil - Do not know |  |
| Cholesterol is found in____________. | *Removed* |  |  |
| Compared to a regular product, a product with the word "light" on the label may be?   - Lower in calories - Lower in fat - Lighter in texture - Lighter in flavour - Lighter in colour - Do not know | *Retained* | Compared to a regular product, a product with the word "light" on the label may be?   - Lower in calories - Lower in fat - Lighter in texture - Lighter in flavour - Lighter in colour - Do not know |  |
| Which of the following contains more fibre based on equal portion size   - Lentils - Corn flakes - Orange juice - Do not know | *Retained* | Which of the following contains more fibre based on equal portion size?   - 125ml (1/2 cup) lentils, boiled - 250ml (1 cup) cereal, corn flakes, Kellogg’s - 250ml (1 cup) 100% orange juice, with pulp - Do not know |  |
| Which of the macronutrients should supply the largest portion of your energy (calorie) intake? | *Removed* |  | *No more emphasis on recommended macronutrient intake* |
| How many calories should a person of your age, activity level, height and weight consume per day? | *Removed* |  |  |
| Generally, how much water should you consume each day? | *Removed* |  | *Validation question* |
| This water should come from:   - Plain water - Foods - Beverages - A combination of the above | *Removed* |  |  |
| Are you aware of any health problems related to an insufficient intake of water?  If yes, What problems are associated with insufficient intake of water? | *Converted to multiple choice question* | Which (if any) of the following is a health problem related to consuming too little water? |  |

**Table 2: Attitude items**

| Attitude questions  Original C-BANKS | Action taken | Attitude questions  C-BANKS short-form | Notes |
| --- | --- | --- | --- |
| How confident do you feel estimating serving sizes of the foods you eat? | *Rephrased* | I feel confident estimating portion sizes of the foods I eat. |  |
| A healthy diet means choosing empty calorie foods less often. | *Revised* | A healthy diet means choosing highly processed foods less often. | *The new CFG recommends limiting highly processed food* |
| Canada's Food Guide is a useful tool for planning my food choices. | *Retained* | Canada's Food Guide is a useful tool for planning my food choices. |  |
| A healthy body weight can be achieved through proper diet and exercise. | *Retained* | A healthy body weight can be achieved through proper diet and exercise. |  |
| Eating food rich in carbohydrates (such as bread, potatoes and rice), makes people overweight. | *Removed* |  | *Addressing misinformation* |
| Recommendations on healthy ways to eat change so often, it's hard to know what to believe. | *Removed* |  | *Opinion question* |
| What you eat can make a big difference in your chances of developing a chronic disease such as heart disease. | *Retained* | What you eat can make a big difference in your chances of developing a chronic disease such as heart disease. |  |
| The things I eat and drink now are healthy, so there is no reason for me to make changes to my diet. | *Removed* |  | *Opinion question* |
| Do you think that your intake (from food and supplements) of the following is: [TOO LOW \| TOO HIGH \| ABOUT RIGHT \| DO NOT KNOW] | *Removed* |  | *Validation question* |
| When reading the food label, how confident are you interpreting the:   - the ingredient list - nutrient claims such as "low fat" or "good source of fiber" - calories per serving? - the number of grams or milligrams of a nutrient (such as sodium) - % Daily Value | *Removed* |  |  |
| Do you think you are at risk for any of the health problems related to:   - Too much fat - Too little fiber - Too much salt - Too little calcium - Too little vitamin D - Too much trans-fat - Too much sugar - Too little water | *Removed* |  | *Risk perception* |

**Table 3: Behavior items**

| Behavior questions  Original C-BANKS | Action taken | Behavior questions  C-BANKS short-form | Notes |
| --- | --- | --- | --- |
| When making food choices how often do you  Limit food high in salt? | *Retained* | To what extent do you:   - Limit foods high in salt |  |
| When making food choices how often do you  Limit butter, hard margarine, lard and shortening. | *Revised* | To what extent do you:   - Limit butter, lard and shortening. |  |
| When making food choices how often do you  Choose a diet with plenty of fruits and vegetables. | *Rephrased* | To what extent do you:   - Eat plenty of fruits & vegetables each day. |  |
| When making food choices how often do you  Limit food high in sugar? | *Retained* | To what extent do you:   - Limit foods high in sugar |  |
| When making food choices how often do you  Choose whole grain products | *Retained* | To what extent do you:   - Choose whole grain products |  |
| When making food choices how often do you  Eat a variety of foods from each of the food groups daily. | *Removed* |  |  |
| When making food choices how often do you  Choose foods low in saturated fat. | *Retained* | To what extent do you:   - Choose foods low in saturated fat |  |
| When making food choices how often do you  Choose brightly colored (dark green and orange) vegetables each day. | *Retained* | To what extent do you:   - Eat a variety of brightly colored vegetable & fruit daily. |  |
| When making food choices how often do you  Choose foods low in trans-fat. | Removed |  |  |
| When making food choices how often do you  Select fish, lean meats and/or poultry. | Revised | To what extent do you:   - Choose lean meats |  |
| When making food choices how often do you  Select plant-based alternatives to meat (e.g. tofu or nuts) | *Removed* |  |  |
| When making food choices how often do you  Consume source(s) of unsaturated fat each day (e.g. canola oil, olive oil, flax seed): | *Rephrased* | To what extent do you:   - Choose healthy fats (e.g. nuts, seeds, vegetable oils). |  |
| When making food choices how often do you  Eat fish two times per week. | *Removed* |  |  |
| When making food choices how often do you  Drink at least two cups milk or fortified soy beverage daily. | *Revised* | To what extent do you:   - Choose lower fat dairy products |  |
| Please rate the importance of the following on your decision to purchase a food item.   - Food safety - Nutritional value - Price - Best before date - Convenience - Occasion - Culture - Health - Organic - Locally produced - Habit - Ease to prepare - Taste | *Revised* | Please rate the importance of the following on your decision to purchase a food item.   - Food safety - Nutritional value - Best before date - Health | *Other options removed because they measure preferences or environmental consciousness instead of health/diet behaviors* |
| When purchasing foods, identify the extent to which you use the following to help you make a decision.   - The ingredient list - The nutrition facts table - Nutrient content (e.g low in fat) - health claims (statements on the food label that describe health benefits of the food or nutrients in the food) - The serving size | *Retained* | When purchasing foods, identify the extent to which you use the following to help you make a decision.   - The ingredient list - The nutrition facts table - Nutrient content (e.g low in fat) - health claims (e. reduced risk of heart disease) - the serving size |  |
| When reading information on the Nutrition Facts table do you look for information about:   - calories - sodium - fat - saturated fat - trans fat - vitamin A - vitamin C - iron - fiber - sugars | *Revised* | When reading information on the Nutrition Facts table do you look for information about:   - calories - sodium - saturated fat - iron - calcium - fiber - sugars | *Vitamins A and C removed from the labels. Canadians are getting enough of these vitamins in their diet* |
| Do you use the Nutrition Facts table to make decisions to purchase:   - dessert items like cookies or cake mixes - Frozen/prepared dinners or main dishes - Breakfast cereals - Cheese - Butter or margarine - Processed meat products like hotdogs or luncheon meats - Snack items like chips or pretzels - Canned foods - Beverages | *Retained* | Do you use the Nutrition Facts table to make decisions to purchase:   - dessert items (cookies, cakes) - Frozen/prepared dinners or main dishes - Breakfast cereals - Cheese - margarine - deli meat - Snack items (chips, pretzels) - Canned foods |  |
| In order to create a healthy diet, indicate the importance of eating the following meals:   - Breakfast - Lunch - Dinner - Snacks | *Removed* |  | *Opinion question* |
| According to Canada's Food Guide recommendations, how many moderate physical activity minutes should be accumulated per day to maintain good health for a person of your age? | *Changed from open-ended to multiple choices* | How many 'sweat a little' to 'sweat a lot' physical activity minutes do you accumulate in a week? |  |
| Would you say that your actual physical activity is greater than, less than, or the same as the recommended number of physical activity minutes for a person of your age? | *Removed* |  | *Validation question* |
| How many calories should a person of your age, activity level, height and weight consume per day? | *Removed* |  |  |
| Would you say your usual energy (calorie) intake is greater than, less than, or similar to your daily energy (calorie) needs? | *Removed* |  | *Validation question* |
| What resources do you usually use to update your nutrition knowledge? | *Removed* |  | *Personal Practice* |
| Which of the following resources would you identify as being reliable sources of nutrition information? | *Removed* |  | *Opinion question* |
| Which, if any, of the following supplements do you take on a weekly basis? | *Removed* |  | *Personal practice* |
